# WISP, Wearable Inertial Sensor for Online Wheelchair Propulsion Detection

**DOI:** 10.3390/s22114221

**Published:** 2022-06-01

**Authors:** Jhedmar Callupe Luna, Juan Martinez Rocha, Eric Monacelli, Gladys Foggea, Yasuhisa Hirata, Stéphane Delaplace

**Affiliations:** 1 Versailles Engineering Systems Laboratory, University of Versailles Saint-Quentin-en-Yvelines, University of Paris-Saclay, 78140 Vélizy, France; eric.monacelli@uvsq.fr (E.M.); stephane.delaplace@uvsq.fr (S.D.); 2 Compagnie Tatoo “Danse Contemporaine Inclusive”, 77185 Lognes, France; dydys1205@gmail.com; 3 Smart Robots Design Lab, Tohoku University, Sendai 980-8579, Japan; yasuhisa.hirata.b1@tohoku.ac.jp

**Keywords:** propulsion gesture, gesture recognition, inertial sensors, manual wheelchair dance, activity assessment

## Abstract

Manual wheelchair dance is an artistic recreational and sport activity for people with disabilities that is becoming more and more popular. It has been reported that a significant part of the dance is dedicated to propulsion. Furthermore, wheelchair dance professionals such as Gladys Foggea highlight the need for monitoring the quantity and timing of propulsions for assessment and learning. This study addresses these needs by proposing a wearable system based on inertial sensors capable of detecting and characterizing propulsion gestures. We called the system WISP. Within our initial configuration, three inertial sensors were placed on the hands and the back. Two machine learning classifiers were used for online bilateral recognition of basic propulsion gestures (forward, backward, and dance). Then, a conditional block was implemented to rebuild eight specific propulsion gestures. Online paradigm is intended for real-time assessment applications using sliding window method. Thus, we evaluate the accuracy of the classifiers in two configurations: “three-sensor” and “two-sensor”. Results showed that when using “two-sensor” configuration, it was possible to recognize the propulsion gestures with an accuracy of 90.28%. Finally, the system allows to quantify the propulsions and measure their timing in a manual wheelchair dance choreography, showing its possible applications in the teaching of dance.

## 1. Introduction

The contribution of disabled performers to dance has recently been recognized and celebrated [1]. This has given legitimacy to disabled dancers and opened a door to artistic physical activity (PA) for more wheelchair users. Wheelchair users have very scanty access to sports, especially due to the limitations set by the intensity and effort required. Manual wheelchair dance (MWD) is a potential artistic activity and sport option, as it is considered a moderate and low-intensity exercise [2,3]. In addition, participation in dance programs is associated with improvements in physical, emotional, and social capacity [4]. However, a significant part of MWD is dedicated to the propulsion. In [2], it was shown that the propulsion movements require up to 30% more time than movements such as raising hands or clapping. In addition, the questionnaires in the study yielded results of fatigue and physical demand during a dance game session, which were almost twice as high in wheelchair users compared to able-bodied players. Gladys Foggea, a French professional dancer and MWD teacher, comments on the need for monitoring in wheelchair propulsion in choreography, “*Because the propulsion in the wheelchair, in analogy to the steps of able-bodied people, must be coordinated with the dance gestures. It is therefore essential to know that propulsions are carried out at the required time.*” Measuring propulsion times is also essential, as Gladys denotes that “*Moving forward, turning and moving on a specific surface in a given time, is a difficult skill to develop during dance training. The number of propulsions must also be quantified, because just like the number of steps taken by a valid dancer, in an established dance choreography there must be a certain number of propulsions in a defined time.*” This leads to the need to monitor MWD for optimization in quantity, timing, and choreographic assessment.

In MWD, propulsion monitoring (PM) is a suitable tool to evaluate time dedicated to propulsion, non-artistic efforts, and meaningful expressions [5]. PM provides important information for the study of an athlete’s performance in sports and rehabilitation progress [6,7]. Roy J. Shephard studied the efficiency of propulsion and the cost of energy compared to gait [8], whereas other biomechanical studies reveal the importance of optimizing propulsion for injury prevention [9,10,11]. Conducting wheelchair dance assessment by mobility and PM also allows the study of capacity improvement [4], mobility, and cognitive learning of wheelchair dancers [1]. In addition, detection of specific gestures in wheelchair dance can define how expressive the dance choreography is [5]. 

Wearable IMU-based systems are widely used for wheelchair physical activity monitoring (WPAM) [12,13,14,15], gesture recognition [16,17,18,19], and performance evaluation of athletes during sports activities and training [20,21,22,23]. Since they are worn for long periods of time, invasiveness and weight influence the user comfort during monitoring. Small, stable, and long-running-time devices are more suitable for users and researchers [24,25]. It has been reported that the attachment of inertial sensors on the upper extremes of the user and on the wheel of the wheelchair allows for estimation of the number of revolutions and the distance traveled by the wheel [26], and authors in [12] also used accelerometers to detect wheelchair propulsion in daily life. In [6], the authors detect self or assisted propulsion by means of inertial sensors during daily life. Another technique for WPAM used in the literature is the self-reported monitoring, which is based on questionnaires and user interviews; however, subjective measures are susceptible to overestimation of data since they must rely on the user’s statements [27,28]. These kinds of assessments do not provide objective data about the user’s activity, such as specific propulsion time or number of propulsions per time. Peter Schantz et al. [29] used electromyography (EMG) to detect upper limb muscle activation in patients with paraplegia and tetraplegia during WP. This method has the advantage of obtaining muscle activity to show importance of trunk movement and technique during propulsion in rehabilitation phases. However, when using EMG, the number of sensors to obtain good accuracy varies with the movement or gesture to be studied, which could lead to a large amount of sensors. Several works investigated the simulated manual WP and session recording for visual feedback [11,30,31]. WP simulations address the aspects of performance improvement, overall experience, and satisfaction [32,33]. While these subjects are relevant to the lifestyles of wheelchair users, and are of great use in PM, a virtual environment simulation station is not always affordable and wheelchair sports, especially dance, tend to be dynamic and require considerably large rooms.

Visual feedback and annotations by session recording is well explored in [10] by using software to display parameters such as push angle, cadence, and velocity on a screen. However, visual feedback normally provides only parameters related to wheel speed and displacement, and specific propulsion gestures (PGs) were not explored. Other methods are limited to detecting the rotation of the wheel by instruments attached to wheelchair structure and logging the daily number of turns [34,35]. Hiremath et al. developed and evaluated a multi-sensor system to detect rest, wheelchair propulsion, arm ergonomics, and desk work. The accuracy of the classifiers reaches 94%. Unfortunately, the system cannot be used in real time because it uses accelerometers, skin conductance sensors, temperature sensors, and a metabolic cart synchronized to the system. In other words, it is a very invasive and complex system [36].

Most of the current PM solutions in wheelchairs do not provide specific information about PGs and the execution time. Furthermore, they are not used in MWD applications. As MWD is currently gaining momentum, it is imperative that monitoring by specific gesture recognition is carried out for assessment in performance and learning. Consequently, this work proposes the utilization of a wearable inertial sensor system for wheelchair propulsion recognition (we named it WISP). Eight PGs, named left-forward, left-backward, right-forward, right-backward, forward, backward, clockwise rotation, and anti-clockwise rotation, and one random movement named dance, are recognized. IMU sensors are attached to the user and linear acceleration and angular velocity are extracted from all axes ‹‹X, Y, and Z››. Sensor readings are classified by a machine learning algorithm to differentiate between wheelchair propulsion and dance movements when performing dance choreography. Our proposed system can recognize predefined user actions using a wearable system based on inertial sensors associated with a classification algorithm. It quantifies specific PGs and it is also able to measure the initial time and duration time of PGs performed by a manual wheelchair dancer during a choreography.

In the following sections of this paper, Section 2 is a description of the proposed system and its design. In Section 3 we explain the data acquisition process. Following this, Section 4 describes the data processing and training of the algorithm. Then, in Section 5, the application case of the system is presented, and, finally, Section 6 presents a discussion of the results which is carried out in order to evaluate the performance of the system.

## 2. WISP

### 2.1. Proposition

**Figure 1:**
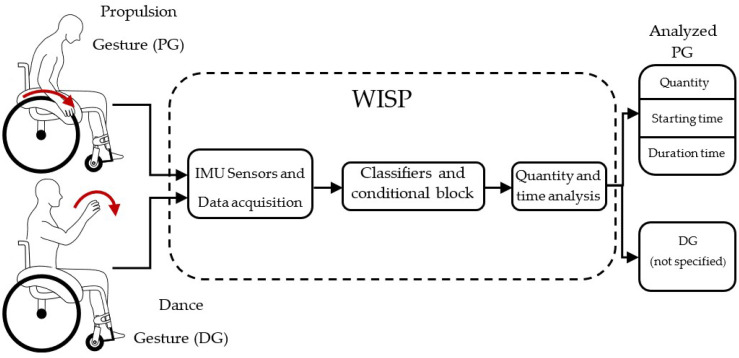
Raw acceleration and angular velocity are read from IMU sensors during propulsion and dance gesture performance. The data extracted are treated and classified by the machine learning classifier algorithm and it provides analyzed data for manual wheelchair dance assessment.

WISP is a new device intended for MWD assessment. The system is based on inertial sensors and it is formulated on two configurations: three-sensor and two-sensor mode. We have considered the possibility that three sensors could better serve gesture detection due to the fact that a sensor on the back could measure the displacement of the person. The configurations using two or three sensors are evaluated in the next sections. Figure 1 shows the scheme for PG recognition. The dancer performs propulsion and dance gestures and these are captured using the inertial sensors. The sensors form a wearable system, as shown in Figure 3. The signals are processed and classified. Subsequently, they are analyzed to obtain the number, duration, and onset time of specific PGs. Anything that is not recognized as PG is considered to be DG. Additionally, the system is developed to work online by means of sliding window process (Section 4.1). 

### 2.2. Hardware Design

**Figure 2:**
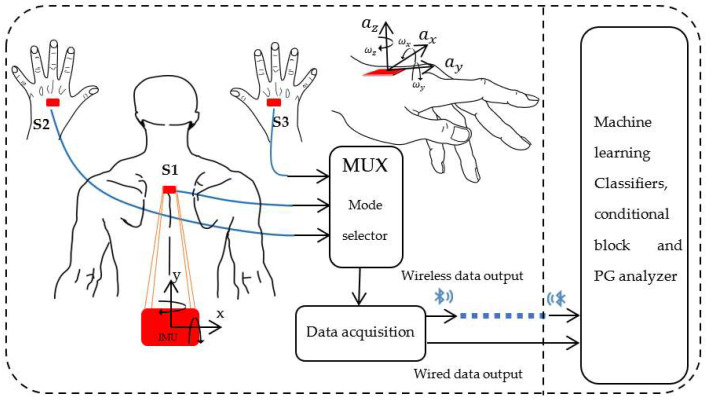
WISP: The left side shows the IMUs placements, the MUX for mode switching, and the data extractor used to extract the raw data. All components on the left side are contained in the wearable part of the system. The right side shows that the wearable part of the system is linked to a processing hardware that uses a classifier to recognize the gestures.

The system consists of three low-cost six-axes (three-axes accelerometer and three-axes gyroscope), IMU (MPU-6050), so that angular velocity and linear acceleration can be extracted on the three axes X, Y, and Z. Within three-sensor mode, IMU S1 is fixed on the back, and IMU S2 and S3 are positioned on the back of the left and right hands respectively, as can be seen in Figure 2. In two-sensor mode, only sensors S2 and S3 are used. The communication between the sensors and the data extractor uses the I2C communication protocol. To read the data from all the IMUs using only one port, an I2C multiplexer (TCA9548A) is added, so that the sensors communicate sequentially one by one with the data extractor. As previously mentioned, WISP will be evaluated in its two modes, so the MUX facilitates the selection between three-sensor (back and hands) and two-sensor (only hands) mode. A second double-mode port of the data extractor is used to send data through a Bluetooth module (HC-05) or through a physical connection. Bluetooth communication is an advantage that allows monitoring from a distance of 15 m without walls in between, which is suitable for the minimum room sizes (10 m × 10 m) according to the National Dance Center in France (CND). This modality facilitates dance assessment because it allows to proceed remotely in the scenario, unlike solutions such as WP simulation platforms [34,35]. In addition it is possible to communicate with devices that display system information and gesture recognition in various ways, such as real-time graphs, performance indicators, scores, etc.

**Figure 3:**
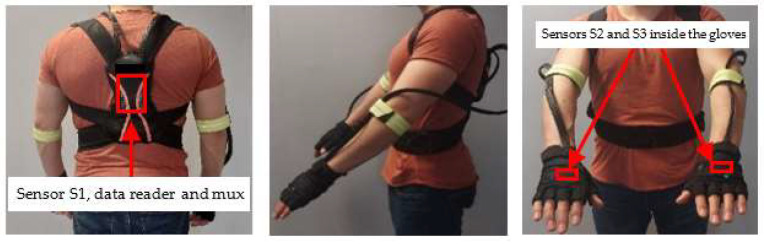
Sensor system and attachment instruments.

Sensor S1 is fixed on the back, the data acquisition hardware and the MUX are fixed together with it, and the attachment was made by means of a back-posture corrector. To attach the IMUs on the hands, they are fixed inside gloves and they are wired along the arms to the back where sensor S1 is located. The fastening of the sensors is illustrated in Figure 3.

### 2.3. Algorithm Structure

**Figure 4:**
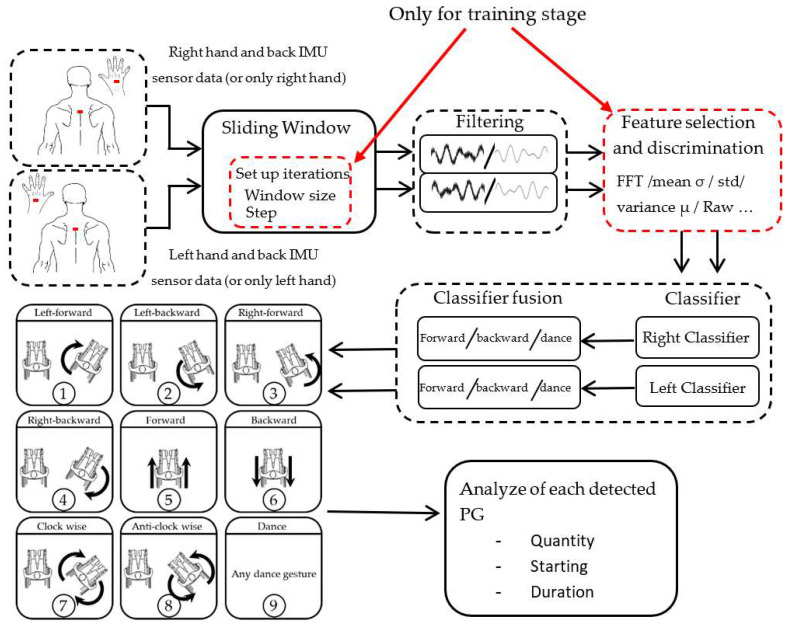
Algorithm structure of WISP.

Raw data from the inertial sensors are acquired and stored by a data processing device. These data were processed using the window sliding method, so that, WISP could be used in online dance assessment. The window sliding method was tuned by iterating the window size and the step. Subsequently, a filtering step was applied, as well as a feature selection block, in order to reduce the number of features used and to avoid a long processing time. Then, data are treated by two bilateral classifiers, assigned to the left and right side of the user. Each of these classifiers recognizes three basic gestures (forward, backward, and dance), each corresponding to its respective side. Subsequently, the outputs of these classifiers are analyzed and fused in order to obtain eight specific PGs in total. Finally, each detected PG was evaluated in order to obtain the number of times it was performed, the start time, and duration of each propulsion. These data provide objective information on MWD performance and will be used by dance teachers. The algorithm structure is shown in Figure 4.

## 3. Data Acquisition

Eight valid subjects were recruited to voluntarily perform MWD choreographies defined by Gladys Foggea. The choreography includes eight defined PGs and DGs. It was repeated 10 times by the subject and the data obtained were logged for further analysis. A data logger was adapted to WISP and it includes an FSR sensor that will be used for hand–rim contact detection only during labeling (Section 3.3). The force sensor (FSR) is attached to the palm of each hand inside the gloves, so that when the palm of the hand touches the rim of the wheel, a signal is received in the data logger from the FSR sensor, indicating such contact. The data logger and FSR were necessary only for data acquisition and labeling work. Once the system is working online, the Bluetooth option will be used and no extra device will be needed. Works on hand gesture reported gesture signal frequencies from 10 Hz to 100 Hz [16,37,38]. Thus, in this study, due to the number of signals extracted, the sensor sampling frequency was set at 30 Hz.

### 3.1. Detected Gestures

The gestures performed during MWD choreography can be classified into two types, propulsion (PG) and dance (DG), according to Gladys Foggea. Hence, all those movements within the choreography that are not recognized as PGs are considered DGs. In this study, importance of being able to distinguish between propulsion and dance lies in quantifying the PGs and the time spent in both types of gestures. Additionally, DGs that can be similar to GP in terms of the movement of the user’s body and arms are being considered. We call this movement fake propulsion gesture (FPG).

#### 3.1.1. Propulsion Gestures

**Figure 5:**
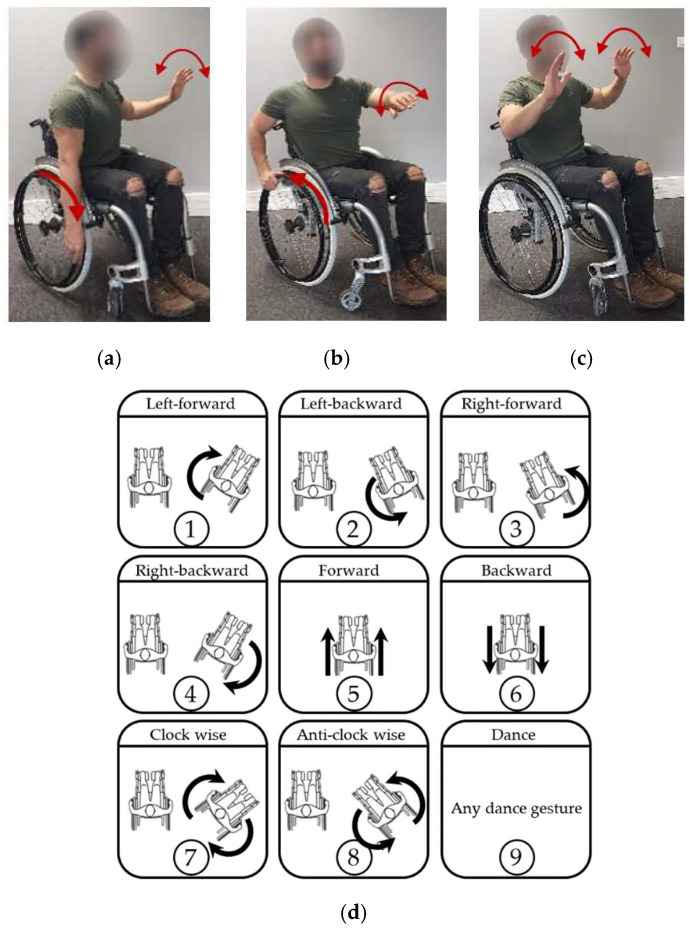
Propulsion and dance gestures: (**a**) “right- forward” gesture, (**b**) “right- backward” gesture, (**c**) dance gesture, and (**d**) all gestures to be recognized.

During wheelchair dance choreography, eight specific PGs will be detected for assessment: left-forward, left-backward, right-forward, right-backward, forward, backward, clockwise rotation, and anti-clockwise rotation. All of them are composed of two basic PGs on each arm. Right hand basic PGs are shown in Figure 5; forward (a) and backward (b). The recognition of PGs will be based on these two movements in both arms, and the remaining PGs are recognized as a composition of them. The ninth gesture shown in Figure 5d will be any of the different DGs addressed in the next section.

#### 3.1.2. Dance Gestures

**Figure 6:**
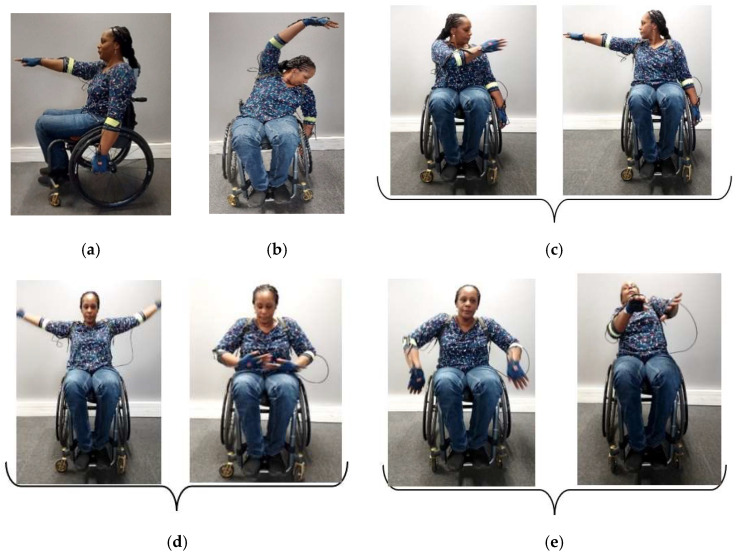
Wheelchair contemporary dance gestures (Gladys Foggea). The gestures are named as follows: (**a**) left/right hand forward, (**b**) left/right arm in curve, (**c**) throwing left/right hand, (**d**) opening and closing, and (**e**) rotation of trunk.

Examples of contemporary dance gestures to be performed during choreography were provided by Gladys Foggea. Figure 6 shows five common wheelchair contemporary dance gestures. Contemporary dance gestures can be enormously varied, however, according to Gladys Foggea; the movements suggested for this work can be taken as a basis for MWD. DGs will not be specifically recognized as in the case of PGs. 

#### 3.1.3. Fake Propulsion Gestures

**Figure 7:**
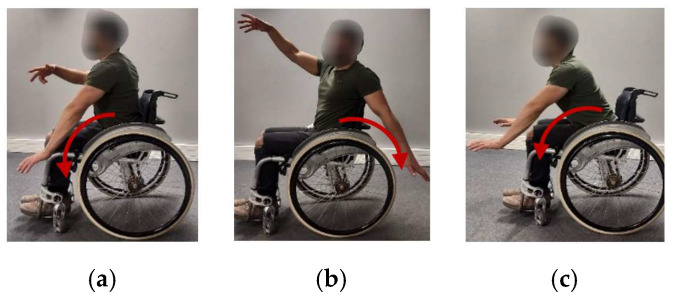
Examples of fake propulsion gestures: (**a**) left-forward, (**b**) left-backward, (**c**) forward.

Given the variety of movements that are performed in MWD, it is common to find movements that are similar to each other. PGs are not exempt from such similarities. It is possible that there are DGs with movements similar to those of propulsion, hence, PG over-detection may occur, which would indicate a poor accuracy of WISP. Thus, for training the recognition algorithm, eight FPGs whose movements are practically the same as the eight PGs studied were considered. As can be seen in Figure 7, the difference between FPGs and actual PGs is that the hands do not propel the wheel when performing FPGs. Thus, FPGs should be classified outside the eight specific PGs, because they are DGs.

### 3.2. Wheelchair Dance Choreography

**Figure 8:**
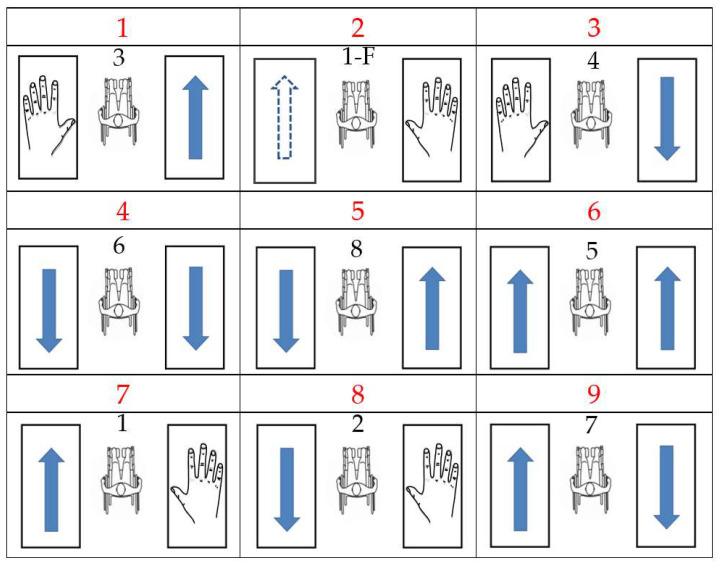
Example of MWD choreography for data acquisition. Propulsion images were presented to the subjects to show what propulsion gesture they must perform. The arrows indicate the arm that will propel and the sense, the hand draw indicates doing a dance movement when only one hand is propelling, and the dot-lined arrow indicates a fake propulsion gesture. Red numbers are the order of the gestures in the choreography.

Each choreography proposed for the experiment is composed of the eight PGs presented in Section 3.1.1. and an FPG taken from those described in Section 3.1.3 (See Figure 8). A different choreography was established for each subject with the nine gestures in random order, and the FPG was also different for each subject. During the choreography, before and after each PG, the users realized the DG mentioned in Section 3.1.2, and even some other DG improvised by the subject. There was no limited number of DG performed between PGs, they ceased once the next PG was due to be performed.

### 3.3. Semi-Automatic Labelization

**Figure 9:**
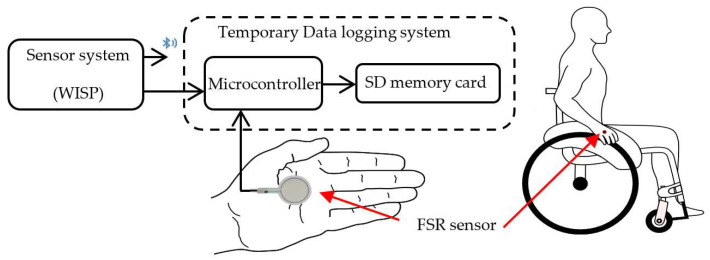
Sensors of WISP are temporary connected to a data logger system. The FSR is added for hand–rim contact detection and only for semi-automatic labeling. FSR serves as reference of PG.

**Figure 10:**
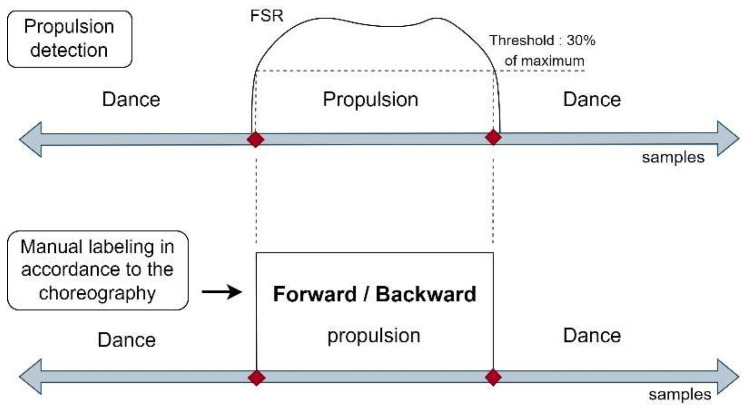
Semi-automatic labeling.

During data acquisition we used the wearable part of WISP (the left part in Figure 2) and a complementary system to log data. In addition, for the semi-automatic labeling, it was necessary to detect when the person had handled the rim to perform the propulsion. Thus, the data logger included one FSR sensor that was temporary placed on each hand, in the area of the palm that has contact with the rim when propelling, as can be seen in Figure 9. In this way, each hand–rim contact was sensed and logged. Then, the signals from the inertial sensors and the hand–rim contact times from the FSR sensor were synchronized. The data collected for each choreography were segmented according to the propulsion times provided by the FSR sensor signal boundaries (Figure 10) and the choreography of each participant. As a final step in the labeling, the sliding windows procedure described in Section 4.1 was used.

## 4. Algorithm Development

The data acquired using our WISP device were saved in an SD card. Thus, one data file was obtained for each trial performed, in which 17 variables were recorded at a frequency f = 30 Hz. The data recorded for each sampling are shown in Table 1.

Ten trials were performed with each of the eight participants. A total of eighty data files were obtained for later processing. The structure of our algorithm proposes the use of two machine learning algorithms: “left classifier” and “right classifier”. The data logger recorded the gesture signals on three-sensor mode (for training phase in two-sensor mode, Sensor S1 can be neglected by programming), so that data inputs for each classifier are going to be from the back and the respective hand, according to the classifier side. Thus, each algorithm will be focused on one side detecting if the person is performing a forward PG, backward PG, or DG. Therefore, from these gestures on each side of the person, it will be possible to detect and know if the person performed any of the eight PGs presented in Section 3.1.1. Consequently, the acquired data will be divided into two datasets, denoted AD (left) and AD (right), which can be expressed as

(1) $$AD_{side}=\overset{P}{\underset{p=1}{⋃}}\overset{T}{\underset{tr=1}{⋃}}\{d_{p,tr,i}\;|\;d_{p,tr,i}\;\in \;R^{N}\;,\;i=1,\;2,\ldots n_{p,tr}\}$$

where *side* indicates the side of the classifier algorithm *side = {left, right}*, and *N* is the number of variables, which depends on the side according to Table 2. $n_{p,tr}$ is the number of extracted samples for each participant p in each of the trial tr and in a trial time t; $n_{p,tr}=f\times t$.

### 4.1. Sliding Window Processing

**Figure 11:**
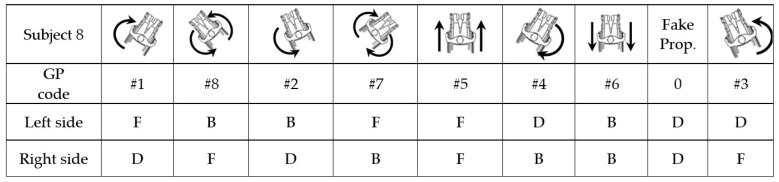
Example of choreography performed by a subject. Left and right classifiers only recognize three gestures for each one, and the rest of choreography are gestures composed of the gestures of both arms. FPGs are recognized as dance gesture. Dance (D), forward (F), and backward (B).

In the previous section it was presented that the data from each trial were divided into two groups of data, so that each classifier could be trained with its corresponding data set. In this way a single classifier should only detect three basic gestures, “right-forward”, “right-backward”, and “dance” for the «right classifier», and “left-forward”, “left-backward”, and “dance” for the «left classifier». In the example of choreography in Figure 11, it can be seen that it is only necessary to recognize the three mentioned gestures with each classifier, since the remaining gestures can be obtained by combining the results of both classifiers. The choreography includes the eight PGs and an FPG, and we remark that non-propulsion gestures and FPGs are considered as a DG. It can be observed that each arm performs three forward propulsions and three backward propulsions.

The signals from the sensors during the propulsion and dance gestures were sectioned with the signals from the FSR sensors in order to perform labeling. To train the classifiers, three forward propulsion movements and three backward propulsion movements from right and left arm present in all the choreographies were extracted as labels. Six random segments of signals of non-propulsion (what we consider as DGs) were also extracted (they include FPGs). In this way, the algorithm was trained to detect gestures with different duration times (between 600 ms and 1500 ms) but strictly delimited. However, this could generate a considerable reduction in accuracy if used in online mode, since the sampling times boundaries are fixed and the PG could be only partially contained in it. For this reason, it was decided to extract the propulsion and dance gestures through the sliding windows process, and the labeling was performed once again with the FSR signal boundary, so that our classifier algorithm can be trained with extracted data in the same way as it would be in an online process.

**Figure 12:**
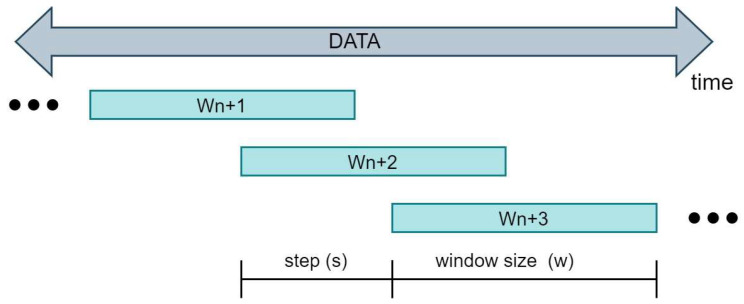
Sliding windows processing.

The sliding window process is frequently used for online data processing and is mainly defined by two parameters: the size of the window and the step. Window size can be understood as the last w samples taken. In addition, the windows are not necessarily consecutive to their borders, but they can overlap. Thus, the next window could start encompassing a certain number of samples before the posterior limit of the predecessor window; this number of samples is known as the sliding step s. Thus, an online system will continuously take groups of data of size w every s samples, and they will be saved for later processing. All of this can be seen exemplified in Figure 12 below.

The data extracted for each test carried out were ordered as shown in Equation (1). Despite the fact that the data were initially saved in their entirety, the sliding windows method will be used to divide each trial into sub-trials that would correspond to each window obtained in a classic online process. Thus, a performed test can be expressed as follows:

(2) $$T=\{d_{i}\;|\;d_{i}\;\in \;R^{N}\;,\;i=1,\;2,\ldots n\}$$

where T is one of the tests carried out, $d_{i}$ is a data sample of dimension *N*, and n is the number of samples taken in the test. Then, the number of windows that would be obtained from each test was initially extracted according to the following formula:

(3) $$m=integer\left(\frac{n-w}{s}\right)+1$$

where m is the number windows per trial, n the number of test samples, w is the predefined window size and expressed in samples, and s is the step chosen for taking the next window expressed in samples.

Thus, each trial could be split into several windows, which had the following form:

(4) $$W_{k}=\{d_{\left(k\times s\right)}\;,\;d_{\left(k\times s\right)+1}\;,\;\ldots ,\;d_{\left(k\times s+w\right)-1}\;,\;d_{\left(k\times s+w\right)}\;|\;d_{i}\;\in \;T\}$$

where  k is the window number which varies from 1 to m.

**Figure 13:**
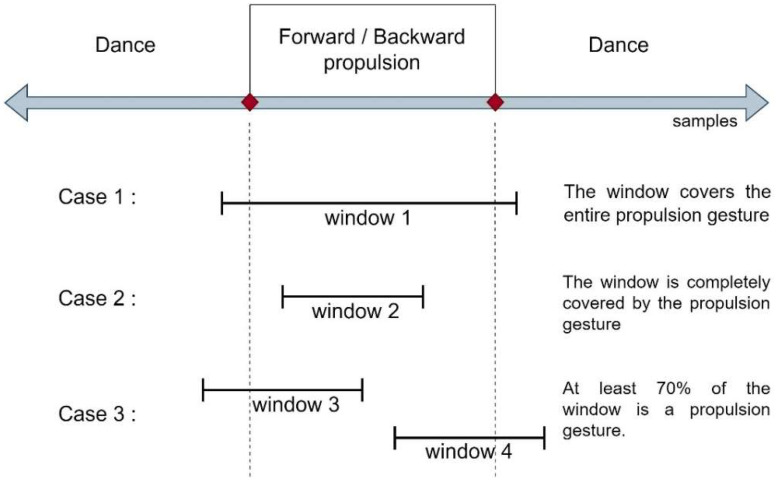
Conditions in the labeling of each window.

Subsequently, each window was evaluated according to its position with respect to the closest propulsion movement, which was provided by the semi-automatic labeling presented in Section 3.3. Thus, the window could receive, as a label, either «propulsion forward», «propulsion backward», or «dance». In this case, the window obtained the label of the nearby propulsion only if it fulfilled one of the three cases presented in Figure 13. In case 1, the label will be set if the window is larger than the launch gesture and completely contains it. In case 2, the window is smaller than the gesture and the gesture contains it completely. In case 3, the window is partially overlapped with the PG. In this case, the label will only be assigned if the PG represents at least 70% of the window size. Finally, in all other cases, the window is labeled as dance.

After each window was labeled, they were grouped so that they could serve as a dataset for the classifier algorithm of the corresponding side. It was observed that the number of windows labeled as dance was much larger than the number of propulsion movements. This was expected because the person performed more random dance movements between each propulsion, and on many occasions the dance time between PGs was up to three times the propulsion time. Likewise, it is necessary to consider that the number of windows labeled as propulsion was greater than those counted by the FSR. This is because each propulsion gesture was frequently covered by several windows at the same time. However, this is beneficial for the algorithm since the input data will be much larger and will be able to achieve better results. Thus, the dataset from a trial, which was processed according to sliding windows and labeled according to the criteria presented above, can be expressed as

(5) $$D_{per\;trial}=\{\left(X_{k},\;Y_{k}\right)\;|\;X_{k}\;\in \;R^{N*w}\;,\;Y_{k}\;\in \;\left\{forward,\;backward,\;dance\right\}\;,\;k=1,\;2,\ldots m\}$$

Finally, this same procedure was carried out for each trial of each participant, as well as for each group of data provided for both classification algorithms. Therefore, the dataset obtained for one of the sides can finally be expressed as

(6) $$Dataset_{side}={⋃}_{p=1}^{P}{⋃}_{tr=1}^{T}\{{\left(X_{k},\;Y_{k}\right)}_{p,t}\;|X_{k}\;\in \;R^{N*w_{p,t}}\;,\;Y_{k}\;\in \;\ldots \;\left\{forward,\;backward,\;dance\right\}\;,\;k=1,\;2,\ldots m_{p,t}\}$$

### 4.2. Classifiers and Features

On *three-sensors* mode, each classifier has ten signals as input, two from the sensor S1 on the back and eight signals from the corresponding sensor on the right or left hand (S2 and S3), whereas on two-sensor mode, each classifier will receive eight inputs coming only from sensors S2 and S3 of the respective hands. Input signal variables for three-sensor and two-sensor are listed in Table 2. Subsequently, based on the yields above, 95% obtained in [39], and those features proposed in the literature [40,41], we proceeded to select a number of $N_{f}$ statistical features for each gesture to be detected. Features in the time domain and in the frequency domain for each of the input data mentioned in Table 2 are shown in Table 3. It is also important to highlight that for the frequency domain features, each datum was preprocessed by a second order Butterworth-type low-pass filter and with a cutoff frequency of 4 Hz.

### 4.3. Parameters Selection and Training

#### 4.3.1. Classifiers and Their Parameters

Several CNN algorithms have been extensively studied in order to increase their accuracy [42,43]. However, due to the size of our dataset [44], we decided to use algorithms that require reduced dataset sizes such as SVM, K-neighbors, and random forest [40]. In addition, for each algorithm, it is recommended to search the hyperparameter space for the best cross-validation score. From the two generic approaches provided by [44], *Grid Search* was chosen as it considers all parameter combinations and gives the best-scoring parameter combination. Iterations were carried out with the K-folds cross validator tool as validator and ten as number of folds. Table 4 shows the parameters values for tuning each algorithm. 

#### 4.3.2. Maximum Number of Features

Based on Table 3, the total number of features to analyze is $N_{f}=19$, considering also the total number of signals to be processed N, analyzing all the signals with each of the features gives us $N\times N_{f}=190$ features for the three-sensor mode and $N\times N_{f}=152$ features for the two-sensor mode. A processing based on a large number of features consumes a large amount of computational time; also, not all features influence the same when classifying. In order to reduce the number and discard irrelevant features, a feature selector was employed [44]. A maximum number of features $N_{fmax}=30$ kept the accuracy of the left and right classifier above 93%.

#### 4.3.3. Sliding Window Parameters Selection

We mentioned above in Section 4.1 that the data obtained for each choreography were processed by the sliding windows method for online gesture recognition. Having already searched for the optimal parameters for the different classifiers and the number of features, we also proceed to evaluate the parameters of the sliding window process as further optimization. This optimization takes place by selecting the appropriate value of window size w and step s. Hence, three w values were evaluated (10, 20, and 30), considering that 30 samples are equivalent to one second, and values 3 and 5 were evaluated as s samples.

### 4.4. Algorithm and Parameters Selection Results

Considering the symmetry of the classifiers, the process provided in Section 4.3 was performed for the right classifier, whose results are shown in Table 5 and Table 6. In this case, the maximum values obtained with the grid search iteration were written for each algorithm.

**Figure 14:**
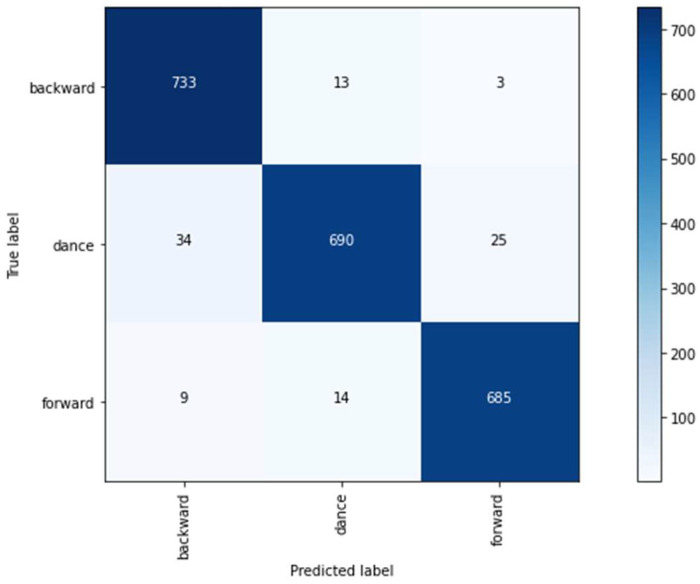
Confusion matrix of right classifier.

From the data analyzed on two-sensor mode, a maximum value of 96.14% was obtained using the random forest algorithm with a window of 30 and a step of 5. On the other hand, on three-sensor mode, a maximum value of 97.43% was obtained using random forest with a window of 30 and a step of 3. This outcome leads us to prescind from three-sensor mode, which means that we can omit the back sensor S1, making our device lighter and more ergonomic. Thus, it was determined that the two-sensor mode would be used for both classifiers. For the right classifier, we used random forest classifier as it provided the highest accuracy for the two-sensor mode. The confusion matrix of the right classifier is shown in Figure 14. In addition, the dataset used for the training of this classifier comes from the sliding windows process and is composed of 749 samples for backward PG, 708 for forward PG, and 749 for DG (this quantity is the maximum between backward and forward to balance the dataset. Samples were taken randomly).

**Figure 15:**
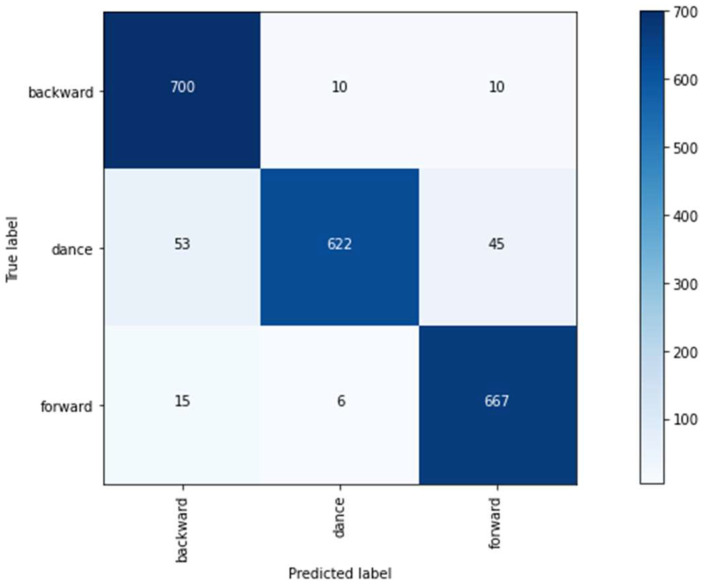
Confusion matrix of left classifier.

Finally, for the left classifier, a window of 30 and a step of 5 was set (the parameters obtained in the iteration for the right hand). In addition, in order to find the most appropriate classifier, the *Grid Search* tool was used again. Thus, we calculated that the left classifier will have a maximum accuracy of 93.91% with the SVM algorithm (kernel = Rbf, C = 10). The confusion matrix of the left classifier is shown in Figure 15. Performed the same way as the right classifier, the dataset used to train this classifier comes from the sliding windows process and is composed of 720 samples for backward PG, 688 for forward PG, and 720 for DG.

### 4.5. Estimation of Propulsion Gestures

As a final step, the outputs of the left classifier and the right classifier were used to reconstruct and estimate the eight performed PGs. This was performed by means of a conditional block whose logic is shown in Table 7. It is important to highlight that a filter was applied in order to eliminate those gestures that were detected for small time lapses (less than 50 ms), which are understood as confusions by the classifiers. Finally, to evaluate the overall accuracy will be the multiplication of the accuracies of left classifier and right classifier in two-sensors mode, which is 90.28%.

## 5. Application of WISP in Wheelchair Dance Teaching

### 5.1. Issues Addressed in Wheelchair Dance Teaching

The propulsions of a wheelchair dancer in choreography have the same purpose as the footsteps of an able-bodied dancer. As a wheelchair dance teacher, Gladys Foggea emphasizes the precision of the steps or propulsions performed. The propulsions are also linked to the rhythm and therefore also to the tempo of the melody. It is necessary that they are carried out in a certain section of the melody. As a case of application of WISP, in the following subsections we will address three essential factors for wheelchair dance assessment according to Gladys Foggea.

#### 5.1.1. Number of Propulsions

**Figure 16:**
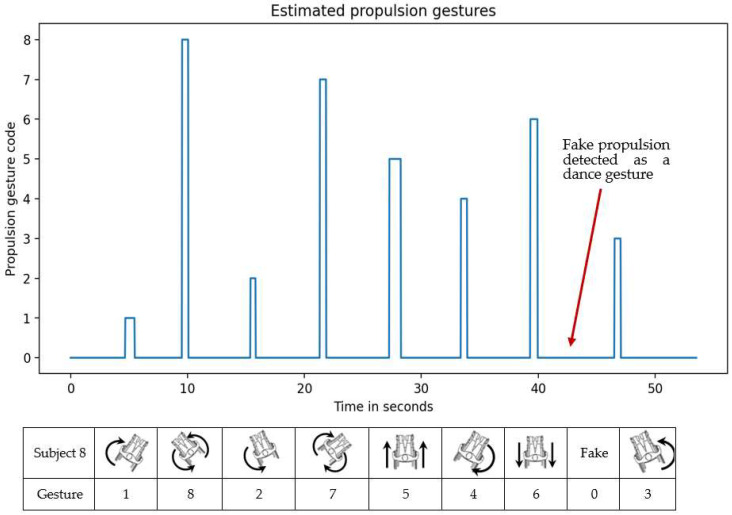
Actual choreography and prediction comparison.

While the propulsion serves the movement of the dancer, such as the leg movements of a valid dancer, propulsions synchronized with the music are considered dance steps. Consequently, in order to execute a choreography, a specific number of steps (propulsions) is required. As already discussed in Section 2, quantifying the propulsions on wheelchair dance steps is the first feature of WISP. Figure 16 shows the prediction results of the system. The predicted propulsions agree in time and quantity and these results can be used as evaluation criteria since the specific gestures can also be displayed.

In Figure 16, it is possible to observe the estimation of the propulsion gestures provided by WISP. Thus, as a first result, WISP is able to provide the number of propulsion gestures performed. In addition, it is possible to see that WISP was able to classify the FPGs as dance, which was envisaged in the training of the recognition algorithms.

#### 5.1.2. Propulsion Starting Time

Propulsion starting time is a key issue in wheelchair dance. Gladys expresses that “*Dance requires attuned movements, but it also requires precision, so propulsions that starts at uncoordinated times with the music or with the planned choreography can make the choreography unaesthetic, even if the correct number of propulsions is performed*”. In addition, regarding the teaching of the precision of the beginning of the propulsion, Gladys adds that “*Precision is a difficult skill to master, and showing students a small but significant difference in propulsion starting time is tricky. Feedback based on propulsion time marks would make it possible to illustrate correct and incorrect performances, thus it would be easier to explain errors of precision*.”

**Figure 17:**
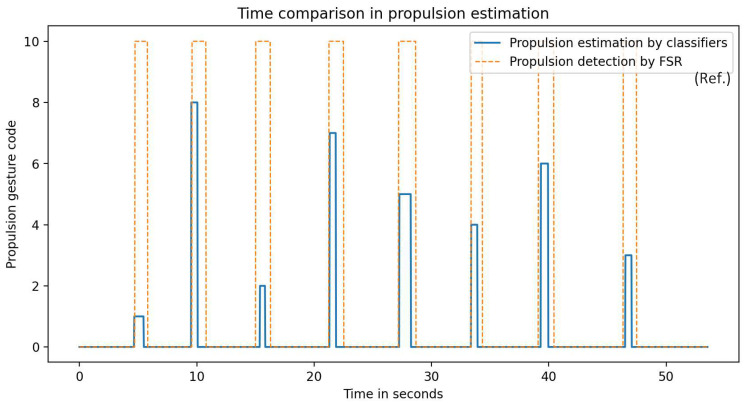
Actual choreography and prediction comparison.

Thus, one of the features added to the WISP algorithm was that it can provide the PG starting time, so that it can be used in wheelchair dance teaching. In addition, in order to corroborate the accuracy of this feature, a comparison was made between the propulsion starting times provided by WISP and the propulsion starting times provided by the FSR sensor in one of the choreographies performed, as can be seen in Figure 17.

Propulsion start values that were extracted by WISP and the FSR are presented in Table 8. From these, we calculated the error in each propulsion performed and the mean absolute error (MAE) of the eight propulsion gestures performed in the choreography selected. The resulting MAE was 123.78 ms, which provides good expectations for the evaluation of wheelchair dance considering that the mean propulsion time is one second.

#### 5.1.3. Propulsion Duration Time

One of the variables necessary for the evaluation of the dance is the duration of the propulsion time. In this regard, Gladys expresses that “*It is not only necessary to perform a certain number of propulsions, but also that they can be of equal or different lengths as required.*”

Thus, as the last data extracted from WISP we have the propulsion duration time. Table 9 shows the propulsion duration data extracted from WISP and the FSR sensor in the selected choreography. In this case, it can be seen that the calculated error has a mean of 47.84%, most likely due to the considerations used in the signal reconstruction (window size, step, sampling rate, etc.).

## 6. Discussion

This paper addresses the need for monitoring of MWD for assessment. Research has been carried out on different methods of monitoring and assessing physical activity in wheelchair users. However, existent solutions are not applicable to MWD and, as in other physical activities monitoring, MWD real-time assessments can be carried out by means of wearable sensors. The proposal of this paper is the design of a wearable inertial sensor for online wheelchair propulsion detection (WISP). The device is intended to allow professional MWD teachers such as Gladys Foggea to perform self-assessments and student evaluations to improve performance. During an MWD choreography, basically two types of movement are performed: propulsion gestures (PGs) and dance gestures (DGs). Based on the premise of the mentioned duality of gestures, WISP was formulated to detect eight specific PGs and, since the system will be used only during MWD choreographies, we have considered all non-propulsion gestures as DGs. Even to improve the accuracy of WISP, DGs whose movement is similar to PGs were considered. Such gestures were called fake propulsion gestures (FPGs). Since the system is based on inertial sensors, two configurations were considered in this work in order to evaluate the system using two or three sensors. In the three-sensor configuration, one sensor is attached to the user’s upper back and one sensor is attached to the back of each hand; in the two-sensor mode, sensors are only attached to the hands. WISP uses two machine learning classifiers (left and right) to bilaterally detect three basic gestures (forward, backward, and dance) performed with each arm.

From the combination of these gestures, eight specific PGs can be obtained. For the detection of the eight PGs, a classifier fusion step must be carried out at the end of the recognition process. The three-sensor mode only provided a 1% improvement to the individual recognition of each classifier. For this reason, the two-sensor mode was chosen, which has a simpler configuration, with fewer sensors and therefore fewer variables to analyze. Therefore, the two-sensor configuration is a lighter version of WISP that offers a free mobility and wireless data transmission. Such an option is better accepted by users and researchers. However, the three-sensor mode could be useful for other body-motion studies. The overall accuracy from the fusion of both classifiers in two-sensor mode was 90.28%. The reconstruction of PGs composed of both hands has several peaks that indicated a confusing detected PG. However, these peaks were easily removed by filtering, leaving the propulsion gestures with a time duration of more than 50 ms.

The WISP algorithm provided data on the quantity and measures of beginning time and duration time of the propulsions performed by one of the participants. In this first analysis, it can be noted that the number of propulsions was correctly detected. This is due to the spike filter previously mentioned. In addition, Figure 17 shows the comparison of the propulsion gestures detected by WISP and those de-detected by the FSR. The results corresponding to the start of propulsion are presented in Table 8, where it can be observed that the mean absolute error is 123 ms. This error is acceptable if we take into consideration that the mean propulsion time is approximately 1 s. In addition, Table 9 shows the results of the propulsion duration time. In this case, the mean error was 47.84%. The high error found in this measurement could be caused by considerations in the signal reconstruction. Future improvements with respect to this measurement will be necessary to obtain reliable data that can be used by the wheelchair dance teacher. 

Finally, this paper presented WISP as a device for recognizing propulsion gestures where special attention is paid to the classification algorithms. The calculation of the experimental accuracy of WISP will be addressed in future research where the propulsion gestures in choreographies designed by the professional dancer Gladys Foggea and performed by MWD students will be evaluated. Another consideration for future research work is an approach towards specific DG recognition by means of the currently proposed system.

## 7. Conclusions

In this study, research of physical wheelchair activity and MWD monitoring was carried out. Current solutions contemplate approaches mostly based on traveled distances, biomechanical efforts, and athletic performance. Gladys Foggea, professional dancer and MWD teacher, stresses the need to monitor MWD for assessments, performance improvement, and teaching, emphasizing the quantification of propulsions, instant of execution, and duration time. Given the scarce applications of physical activity monitoring in MWD, in this work we developed a wearable inertial sensor for online wheelchair propulsion detection (WISP). This device uses two machine learning classifiers for bilateral detection of propulsion gestures. Furthermore, the device was evaluated in its two configurations: three-sensor and two-sensor. The two-sensor configuration was chosen since it had only 1% lower accuracy than the other configuration. The fusion of the classifiers gave results showing an accuracy of 90.28%. Finally, we conclude that the algorithm of WISP allowed to quantify the propulsions and identify the start instant with a mean absolute error (MAE) of 123.75 ms, as well as the propulsion duration with a mean error of 47.84%.

## Figures and Tables

**Table 1:** Total variables recorded in each trial.

Variables	
Device	Axis
Trunk accelerometer	Z
Trunk gyroscope	Y
Left-hand accelerometer	X
	Y
	Z
Left-hand gyroscope	X
	Y
	Z
Right-hand accelerometer	X
	Y
	Z
Right-hand gyroscope	X
	Y
	Z
Sampling time	-
Left-hand force sensor ^1^	-
Right-hand force sensor ^1^	-

^1^ Data from hand force sensors are extracted and used just for labeling. They are not used to train our machine learning models.

**Table 2:** Input variables for each classifier in both modes: *three-sensor* and *two-sensor*.

Classifier Side	Three-Sensor Mode		Two-Sensor Mode	
	Device	Axis	Device	Axis
Left Classifier	Trunk accelerometer Trunk gyroscope	Z X	- -	- -
	Left-hand accelerometer	X Y Z	Left-hand accelerometer	X Y Z
	Left-hand gyroscope	X Y Z	Left-hand gyroscope	X Y Z
	Left-hand accelerometer norm Left-hand gyroscope norm	|a| |ω|	Left-hand accelerometer norm Left-hand gyroscope norm	|a| |ω|
Right Classifier	Trunk accelerometer Trunk gyroscope	Z X	- -	- -
	Right-hand accelerometer	X Y Z	Right-hand accelerometer	X Y Z
	Right-hand gyroscope	X Y Z	Right-hand gyroscope	X Y Z
	Right-hand accelerometer norm Right-hand gyroscope norm	|a| |ω|	Right-hand accelerometer norm Right-hand gyroscope norm	|a| |ω|

**Table 3:** Features computed for each data; $N_{f}=19$.

Domain	Feature
	Mean
	Rms
	Variance
	Standard deviation
	Median
	Maximum
Time	Minimum
	Zero crossing
	Number of peaks
	25th Percentile
	75th Percentile
	Kurtosis
	Skew
	Number of peaks
	PSD Mean
Frequency	PSD rms
	PSD median
	PSD standard deviation
	PSD entropy

**Table 4:** Parameters values for tuning ML classifiers.

Algorithm	Parameter	Grid Search Values
SVM	Kernel	Linear, Rbf
	C	0.1, 0.3, 0.6, 1.0, 3, 6, 10
K-neighbors	Number of neighbors	3, 5, 10, 15, 20, 40
	Weights	Uniform, distance
	Algorithm	auto, ball tree, kd tree, brute
Random Forest	Number of estimators	50, 100,200
	Criterion	Gini, Entropy
	Max depth	5, 8, 11, 14
	Max features	Auto, Sqrt, Log2

**Table 5:** Maximum values obtained for the right classifier in two-sensor mode.

Results with Hand Sensor						
Algorithm	W = 10		W = 20		W = 30	
	S = 3	S = 5	S = 3	S = 5	S = 3	S = 5
SVM	0.9393	0.9396	0.9499	0.9472	0.9600	0.9438
K-neighbors	0.9302	0.9259	0.9347	0.9254	0.9415	0.9138
Random forest	0.9518	0.9423	0.9537	0.9518	0.9572	0.9614

**Table 6:** Maximum values obtained for the right classifier in three-sensor mode.

Results with Hand and Back Sensors						
Algorithm	W = 10		W = 20		W = 30	
	S = 3	S = 5	S = 3	S = 5	S = 3	S = 5
SVM	0.9457	0.9410	0.9558	0.9502	0.9652	0.9511
K-neighbors	0.9378	0.9165	0.9446	0.9376	0.9492	0.9354
Random forest	0.9515	0.9500	0.9614	0.9556	0.9743	0.9578

**Table 7:** Propulsion gesture estimation from classifier predictions.

Detected Gesture by Classifiers		Estimated Propulsion Gesture		
Left Classifier	Right Classifier			
Forward	Dance	#1	Left-forward	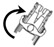
Backward	Dance	#2	Left-backward	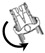
Dance	Forward	#3	Right-forward	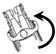
Dance	Backward	#4	Right-backward	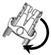
Forward	Forward	#5	Forward	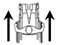
Backward	Backward	#6	Backward	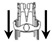
Forward	Backward	#7	Clockwise	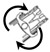
Backward	Forward	#8	Anti-clockwise	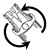
Dance	Dance	#9	Any dance gesture (including FPG)	-

**Table 8:** Propulsion starting time results from WISP and force sensors.

Propulsion Starting Time				
Gesture	FSR (ms)	Classifiers (ms)	Error (ms)	MAE (ms)
1	4740	4680	−60	123.75
8	9600	9540	−60	
2	15,030	15,390	360	
7	21,270	21,330	60	
5	27,210	27,270	60	
4	33,390	33,390	0	
6	39,120	39,330	210	
3	46,350	46,530	180	

**Table 9:** Propulsion duration time results from WISP and force sensors.

Propulsion Duration Time				
Gesture	FSR (ms)	Classifiers (ms)	Error (%)	Mean Error (%)
1	1080	810	25.00	47.84
8	1200	540	55.00	
2	1260	450	64.28	
7	1260	540	57.14	
5	1470	990	32.65	
4	960	540	43.75	
6	1320	630	52.27	
3	1140	540	52.63	

## Data Availability

The data presented in this study are available on request from the corresponding author. The data are not publicly available due to privacy issues.
